# Finite element mechanical analysis of ipsilateral approach and contralateral approach in unilateral bilateral endoscopic spine surgery

**DOI:** 10.1186/s13018-023-04476-z

**Published:** 2023-12-20

**Authors:** Wenzheng Li, Junjian Han, Qingyun Xin, Qitao Liu, Chao Feng, Yichan Liu, Dengjun Zhang

**Affiliations:** 1 Department of Orthopaedics, The Fifth Clinical Medical College of Shanxi Medical University, Taiyuan, 030012 Shanxi China; 2 Shanxi University of Chinese Medicine, Taiyuan, 030024 Shanxi China

**Keywords:** Unilateral bilateral endoscopy, Lumbar herniated disc, Contralateral approach, Finite element method, Spine

## Abstract

**Background:**

Unilateral bilateral endoscopic spine surgery (UBE) is often performed to treat lumbar spinal stenosis and disc herniation. It has become a prominent method in endoscopic spine surgery because of its very low learning curve and broader operative field of vision. Currently, the ipsilateral approach and contralateral approach have been established for disc herniation in the foraminal area, intervertebral foramen region, or pedicle region. The contralateral method offers many benefits over the ipsilateral approach, including less bone labour during microsurgical decompression and the preservation of facet joints. However, because it uses the interlaminar window approach, it inevitably involves osteotomy of the patient’s superior and inferior articular processes, which may result in corresponding deterioration in the spine’s biomechanical stability and subsequent adjacent facet joint diseases caused by facet joint degeneration postoperatively.

**Objective:**

As a result, the purpose of this work is to use a finite element model to evaluate how the ipsilateral approach and contralateral approach in unilateral bilateral endoscopic spine surgery affect spinal stability while treating identical intervertebral disc herniation.

**Study design:**

In this study, a three-dimensional lumbar-sacral spine model was built and verified. Osteotomies were conducted for armpit-type lumbar disc herniation (LDH), periradicular-type LDH, and shoulder-type LDH. Postoperative lumbar spine models of the ipsilateral approach and contralateral approach in unilateral bilateral endoscopic spine surgery were developed. The von Mises stress on the endplate, shear force on the annulus fibrosus, pressure inside the intervertebral disc, and range of motion (ROM) of the L3 segment were all determined. The results of our well-validated model showed that osteotomy done in the ipsilateral approach deteriorated most biomechanical metrics.

**Results:**

In the majority of loading conditions, the contralateral approach caused the intervertebral disc’s biomechanical properties to increase, and the ipsilateral approach caused the intervertebral disc’s biomechanical properties to increase sharply more than the contralateral approach.

**Conclusion:**

The contralateral approach, which is now extensively employed in unilateral bilateral endoscopic spine surgery, may be regarded as an ideal surgical alternative for treating lumbar disc herniation without producing iatrogenic instability. This approach has a low facet joint reduction rate, minimum soft tissue injury, and precisely identifies the midline of the central spinal canal during the retraction of the thecal sac and nerve roots.

## Introduction

The increased human lifetime has increased the prevalence of spinal diseases [1]. Lower back pain and leg problems afflict 1–5% of the population each year, with lumbar disc herniation being the most common cause [2]. Conservative therapy may partly improve symptoms of lower back pain and leg discomfort in persons with mild to severe symptoms [3]. However, discectomy has become an excellent therapy choice for individuals with surgical indications [4]. Endoscopic spine surgery has been increasingly employed in the treatment of lumbar disc herniation [5].

**Fig. 1:**
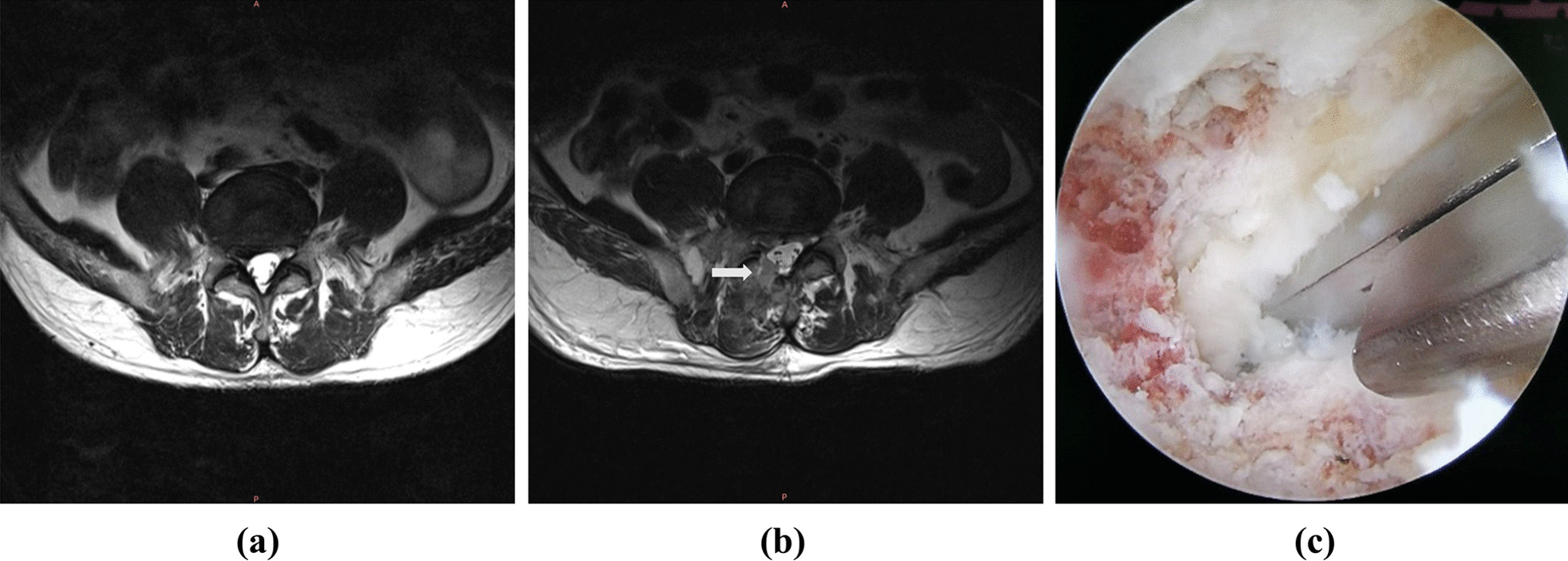
Preoperative and postoperative MRI and intraoperative images of the patient. **a** Preoperative magnetic resonance axial image of the patient showing a herniated lumbar disc compressing the dural sac. **b** Postoperative magnetic resonance axial image of the patient showing the patient’s occluded bone. **c** Intraoperative endoscopic surveillance of the occluded bone

Unilateral bilateral endoscopic (UBE) spine surgery is being used more often to treat lumbar spinal stenosis and lumbar disc herniation (LDH). UBE surgery employs two channels, resulting in a larger and clearer surgical area as well as enhanced operability. These benefits enable surgeons to perform more accurate and comprehensive intervertebral space decompression [6]. Because UBE surgery is commonly performed through the interlaminar route, resection of the facet joints is unavoidable. It has been shown that facet joint resection and osteotomy are related to spinal stability [7] (Fig. 1).

Because of the size of the interlaminar window, direct removal of the disc with a functioning cannula is not possible. As a result, osteotomy is an inescapable surgical strategy in the treatment of lumbar spinal stenosis and disc herniations in the foraminal area, intervertebral foramen region, or pedicle region [8]. In terms of facet joint injury, the recently adopted contralateral approach in UBE surgery for decompression has demonstrated benefits over the standard ipsilateral approach. The ipsilateral approach procedure means that the surgeon stands on the side of the herniated lumbar disc, and the contralateral approach means that the surgeon stands on the opposite side of the herniated disc. If the patient has a right-sided disc herniation, the surgeon should stand on the patient’s left side when performing a contralateral approach from the left side (contralateral) down through the sublaminar space to the right side (ipsilateral) [9]. The contralateral approach has the following advantages: (1) minimal removal of the facet joints, preserving them to a greater extent, and (2) less traction on the nerve roots, resulting in less nerve root damage than the ipsilateral approach [10].

Previous research has shown that the contralateral approach in UBE surgery achieves lower facet joint reduction rates, less soft tissue damage, and less intraoperative traction on the dural sac and nerve roots than the ipsilateral approach for shoulder-type lumbar disc herniation at the same segment [11]. However, there is presently no research describing the size of these two treatments’ postoperative influence on spine biomechanics. Establishing a finite element model of the spine, modelling both techniques and quantifying the biomechanical impacts on the spine to study postoperative outcomes are a solid strategy for assessing these alterations.

To verify this hypothesis, we created models of three types of lumbar disc herniation in healthy adults and performed intervertebral disc decompression using the ipsilateral and contralateral approaches, to prove that the contralateral approach has a lower impact on the biomechanical characteristics of the lumbar spine than the ipsilateral approach.

## Materials and methods

### Model construction

Based on lumbar CT data from a healthy 25-year-old male volunteer with no history of lumbar spine disease, a vertebral model from L3 to L5 was built. A rebuilt cortical shell (0.8 mm thick), trabecular bone, vertebral arches, pedicles, spinous processes, and other components were incorporated into the bone structure [12], assigning values to bone tissue based on the data in Table 1 [13].

**Table 1:** Material properties of spine structures

Structure	Young’s modules (MPa)	Poisson ratio
Cortical	12,000	0.30
Cancellous	100	0.20
Cartilages	10	0.4
Annulus	4.2	0.45
Endplates	24	0.25
Nucleus	1	0.49

Non-osseous structures included the reconstructed annulus fibrosus, nucleus pulposus, articular cartilage of the facet joints, and intervertebral ligaments. The intervertebral disc consisted of the nucleus pulposus, annulus fibrosus, and upper and lower vertebral endplates. The structure of the annulus fibrosus and endplates occupied 95% of the cross-sectional area of the vertebral body. The distance from the front edge of the annulus fibrosus to the back edge of the vertebral body was set at a ratio of 1.62. The positions of these structures were fixed. ANSYS software was used to create the anterior longitudinal ligament, posterior longitudinal ligament, ligamentum flavum, supraspinous ligament, and joint capsules, and material properties were given to these structures [8, 14, 15], assigning values to the ligaments based on the data in Table 2 [16, 17].

**Table 2:** Ligament material properties of the FEA model

Ligament	Young’s modules (MPa)	Cross-sectional areas (mm^2^)	Poisson ratio	Stiffness (kg/m^2^ s^2^)
ALL	7.8	22.4	0.3	8.74
PLL	10.0	7.0	0.3	5.83
LF	17.0	14.1	0.3	15.38
ITL	10.0	0.6	0.3	0.19
ISL	10.0	14.1	0.3	1.85
SSL	8.0	10.5	0.3	2.39
JCL	7.5	10.5	0.3	15.75

ALL: Anterior longitudinal ligament; PLL: posterior longitudinal ligament; LF: ligamentum flavum; ITL: intertransverse ligament; ISL: interspinous ligament; SSL: supraspinous ligament; and JCL: joint capsule ligament

### Simulation of unilateral bilateral endoscopic surgery (UBE)

**Fig. 2:**
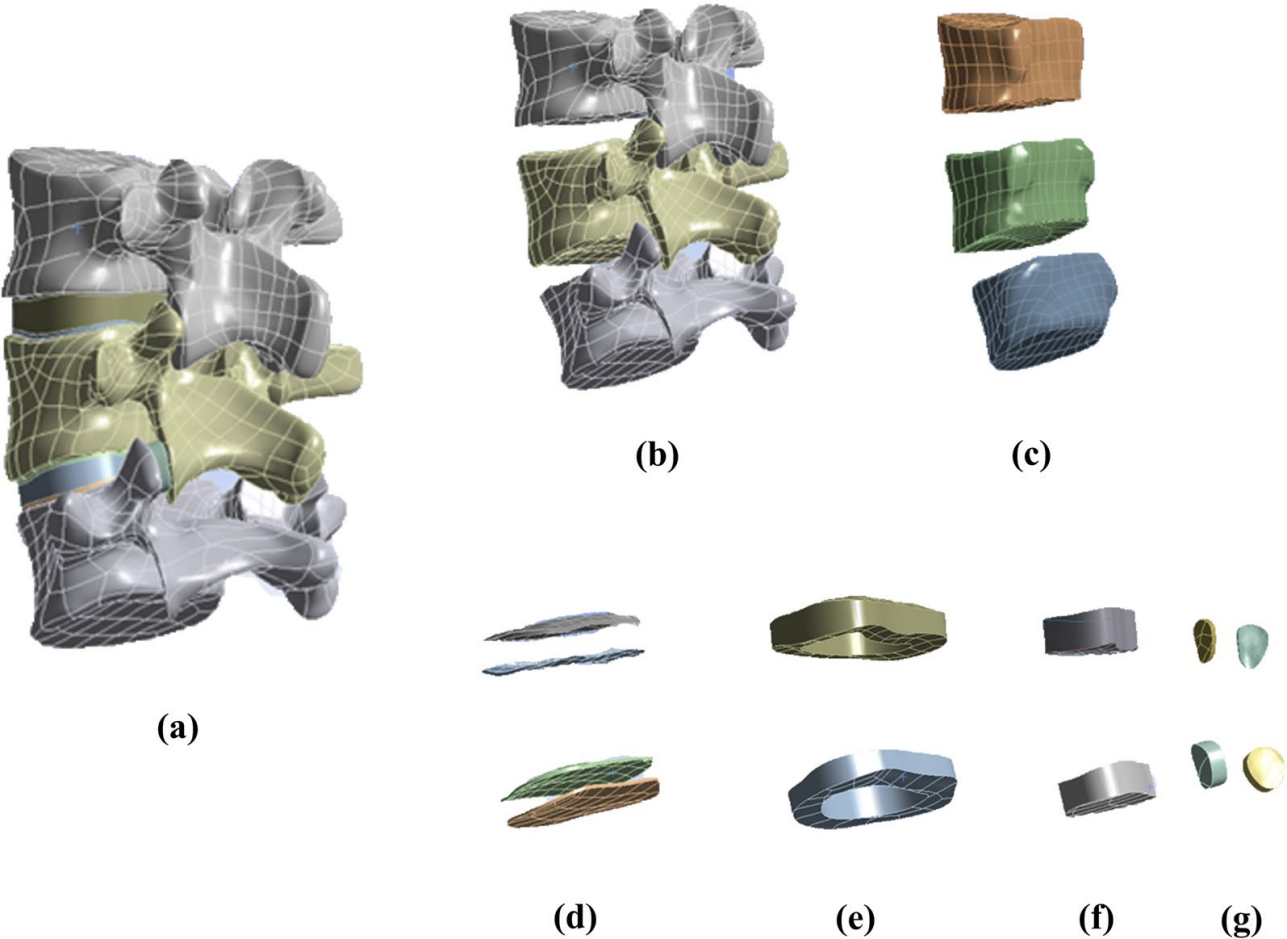
The FEM developed in this study. **a** Lumbar spine 3–5 model. **b** Cortical. **c** Cancellous. **d** Endplates. **e** Annulus. **f** Nucleus. **g** Cartilages

**Fig. 3:**
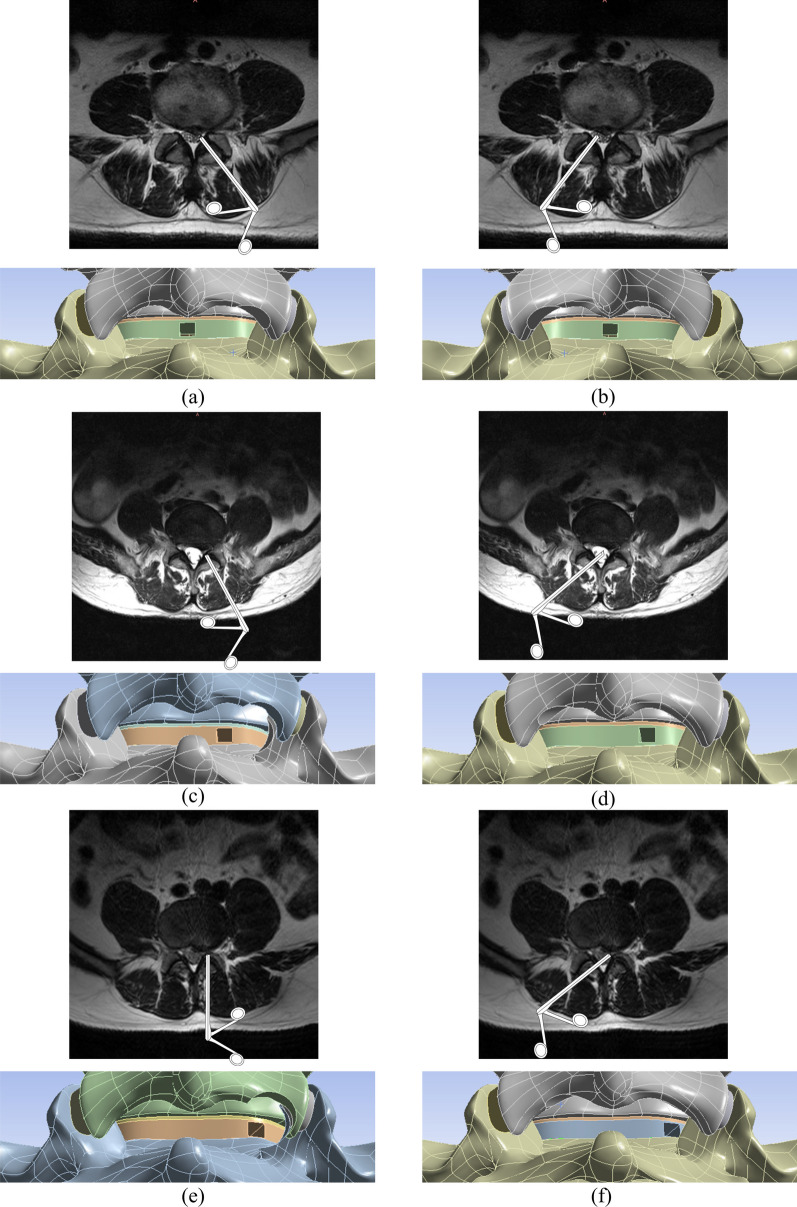
The schematic of the surgical. **a** Ipsilateral approach surgical excision of armpit-type LDH. **b** Contralateral approach surgical excision of armpit-type LDH. **c** Ipsilateral approach surgical excision of periradicular-type LDH. **d** Contralateral approach surgical excision of periradicular-type LDH. **e** Ipsilateral approach surgical excision of shoulder-type LDH. **f** Contralateral approach surgical excision of shoulder-type LDH

The UBE surgery simulation was based on relevant literature and our clinical surgical experience [18]. L4–L5 was chosen as the target segment for discectomy because it is the most usually afflicted section in lumbar disc herniation [19]. The discectomy was done in the ipsilateral approach in the right L4–L5 intervertebral area, directly targeting the protruded disc on the right side. The discectomy was conducted in the contralateral approach at the left L4–L5 intervertebral area, directly targeting the protruded disc on the right side. In our operating room, the arthroscopic cannula was 6 mm in diameter. As a result, we defined the diameter of the lamina forceps as 8 mm, which is 2 mm greater than the diameter of the lamina forceps in all directions, making a standard circle, depending on the surgeon’s competence. The articular cartilage inside the facet joints was also removed during the excision of the facet joints. To duplicate the surgical approach as precisely as feasible, a 4 mm incision was created on the afflicted annulus fibrosus, and one-third of the nucleus pulposus was excised [20]. Figure 2 shows the finished model, and the schematic of the surgical is shown in Fig. 3.

### Boundary and loading conditions

In order to improve the accuracy of the calculations, we set the mesh in the model to a tetrahedral mesh with an articular cartilage mesh size of 0.5 mm and the rest of the mesh mass of 0.8 mm [8, 21]. The model’s surfaces were all specified as frictionless. A vertical compression force of 500 N was applied to the top surface of the L3 vertebrae to simulate the weight of the upper half of the body. In addition, a moment of 10 N m was applied to the surface of the L3 vertebral body during flexion, extension, left lateral axial rotation, right lateral axial rotation, left lateral bending, and right lateral bending to simulate the moments exerted on the spine during the six states of motion of a normal adult [22]. During these six loading phases, the maximum load for each model was obtained, and the bottom surface of L5 was entirely fixed in all directions. The maximum von Mises stress in the upper and lower endplates, maximum shear force in the annulus fibrosus, intradiscal pressure at L4–L5, and vertebral range of motion at L3 were the parameters to be measured [8, 23].

## Results

### Model validation

**Fig. 4:**
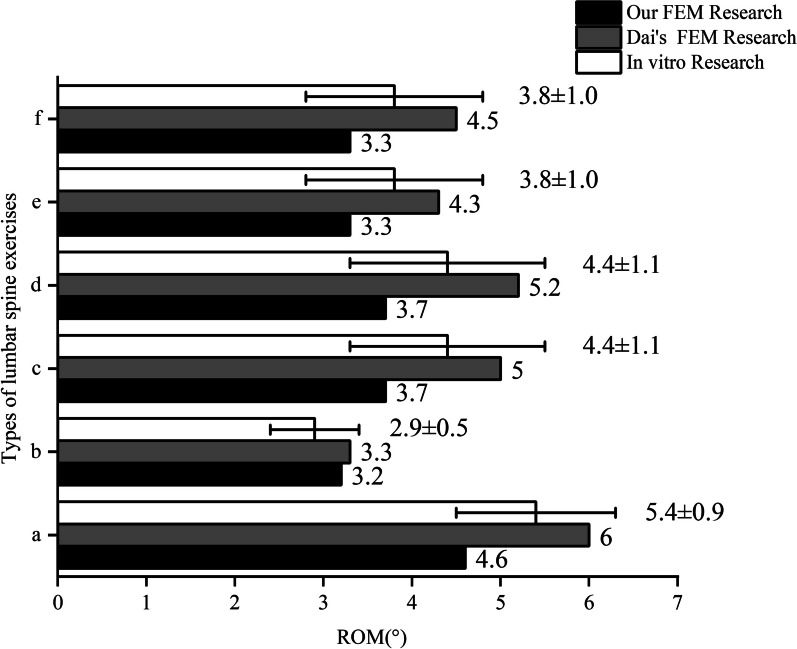
Validation of the FEM model. **a** Flexion. **b** Extension. **c** Left axial rotation. **d** Right axial rotation. **e** Left lateral bending. **f** Right lateral bending

We compared the mechanical data produced from our model with mechanical data from past laboratory cadaver studies to further evaluate the usefulness of our study model. To replicate flexion, extension, lateral bending, and axial rotation motions, the model was preloaded and exposed to pure torque. The mistakes in our model’s range of motion data were within the range of experimental data errors reported by Shim [24], and also, our model is compared with finite element model data from previous studies [25], demonstrating that our experimental model is appropriate for this investigation. Figure 4 displays the findings.

### Variation of biomechanical characteristics

**Fig. 5:**
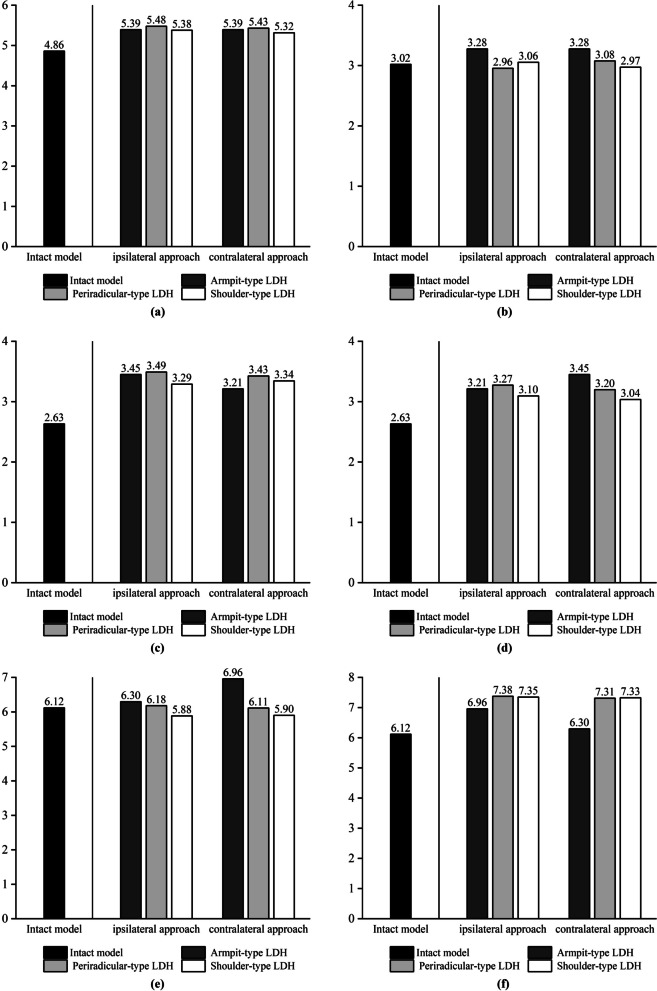
The histograms of variation of force on the upper endplates on L4–L5. **a** Flexion. **b** Extension. **c** Left axial rotation. **d** Right axial rotation. **e** Left lateral bending. **f** Right lateral bending

**Fig. 6:**
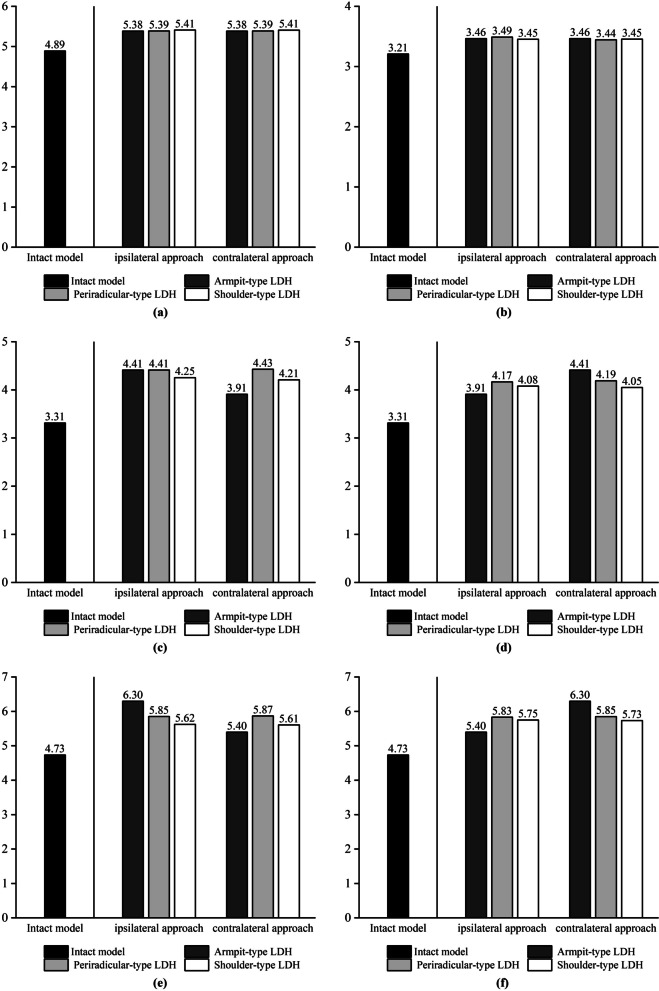
The histograms of variation of force on the lower endplates on L4–L5. **a** Flexion. **b** Extension. **c** Left axial rotation. **d** Right axial rotation. **e** Left lateral bending. **f** Right lateral bending

**Fig. 7:**
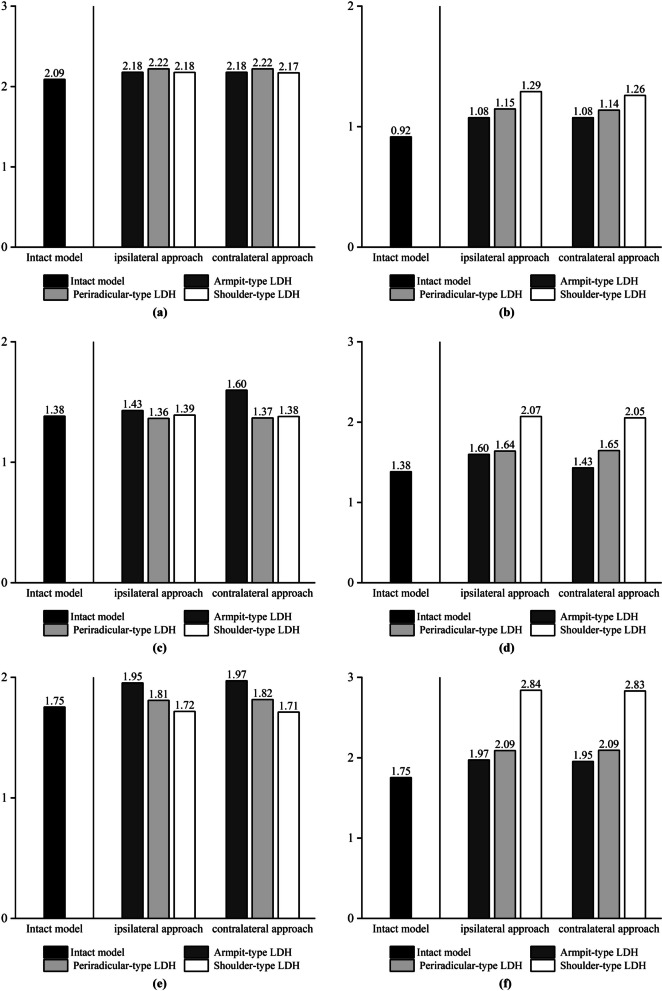
The histograms of variation of force on the annulus on L4–L5. **a** Flexion. **b** Extension. **c** Left axial rotation. **d** Right axial rotation. **e** Left lateral bending. **f** Right lateral bending

**Fig. 8:**
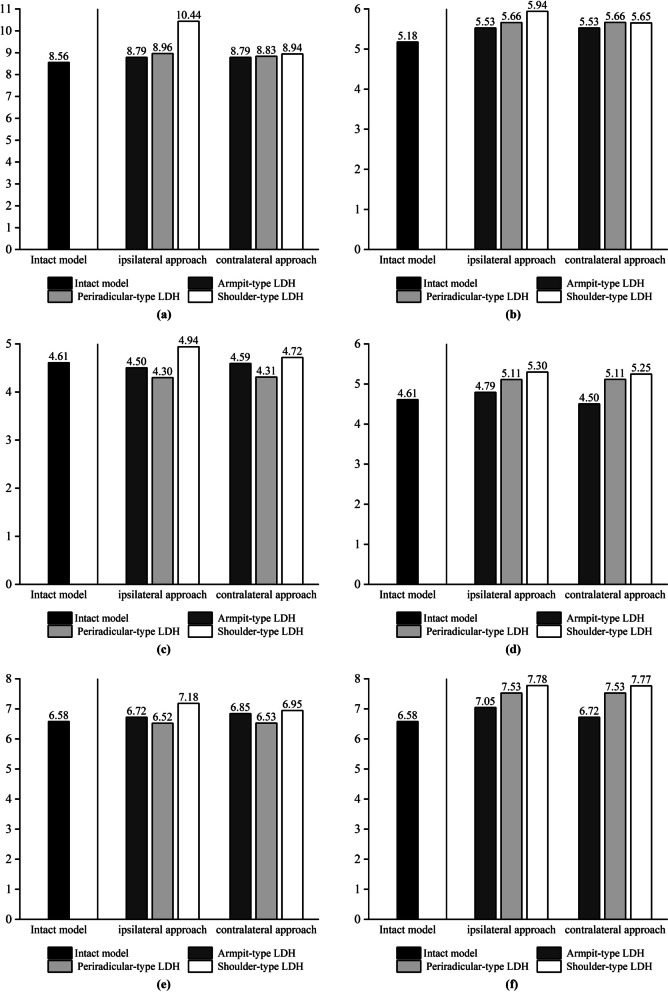
The histograms of variation of ROM on L3–L4. **a** Flexion. **b** Extension. **c** Left axial rotation. **d** Right axial rotation. **e** Left lateral bending. **f** Right lateral bending

We chose the mobility of L3–L4 to assess the ROM value and did not assess the ROM of L4–L5 because the lower surface of L5 is fixed, and assessing the ROM of this segment would result in a low resultant value. We chose the maximum von Mises force of the upper endplate, the maximum von Mises force of the lower endplate, the maximum shear force of the annulus fibrosus, and the intradiscal pressure in the operated segments L4–L5 [8], and we applied these four metrics to assess biomechanical changes in the L4–L5 intervertebral discs. According to our experimental results, the mechanical indicators in the simulated spine model following UBE surgery increased when compared to the intact model. The postoperative model showed an increase in stress on the annulus fibrosus in all motion states compared to the preoperative state, but in left lateral bending, the maximum von Mises stress on the annulus fibrosus in the shoulder-type LDH model decreased compared to the preoperative state, but in right lateral bending, the maximum stress on the annulus fibrosus in the shoulder-type LDH model increased by 62%. The change in mobility of the shoulder-type LDH treated with the ipsilateral approach showed a significant increase compared to the preoperative period, with a 22% increase in forward flexion and a 15% increase in posterior extension movements compared to the preoperative period in vertebral mobility. The maximal stress on the annulus of fibres in the model of shoulder-type LDH treated with the contralateral approach, on the other hand, increased less throughout the preoperative time, with a 4% rise in forward flexion and a 9% increase in posterior extension movements. Mechanical indications in the model of the ipsilateral approach in UBE surgery were poorer than those in the model of the contralateral approach. When treating shoulder-type lumbar disc herniation (LDH) using the UBE method, the mechanical indications were poorer in the ipsilateral approach compared to the contralateral approach. The experimental data are presented in the form of histograms, as illustrated in Figs. 5, 6, 7 and 8.

## Discussion

This study’s design was heavily influenced by two prevalent surgical methods in UBE surgery. However, only clinical trials have validated the improved surgical results of the contralateral technique over the ipsilateral method [11, 26]. Park et al. [11] noted that the herniated disc could be successfully removed with minimal disruption of the articular facets through the contralateral approach, and another advantage of this procedure was that the midline of the central spinal canal could be more accurately identified through the contralateral approach. Yeom et al. [27] suggested that the contralateral approach allowed endoscopic access to the lesion site at a wider angle in order to remove the distally. Hwang et al. [28] reported that the contralateral approach allows for a better understanding of the pathology of the lateral saphenous fossa and maintains spinal biomechanical stability by preserving the facet joints as much as possible, as compared to the ipsilateral approach. All of these clinical studies have demonstrated that the contralateral approach minimizes muscle damage and preserves structures such as ligaments, maximizes the preservation of the facet joints, and avoids compromising the biomechanical stability of the spine. Our study corroborates this view while taking a biomechanical viewpoint that we created models for both ipsilateral and contralateral approaches in UBE surgery using a typical lumbar finite element model. The model was then subjected to finite element mechanical analysis to assess the forces on the intervertebral discs of the LDH model through both surgical treatments.

To begin, there was no statistically significant difference (*p* > 0.05) between the two sets of data, namely the ipsilateral and contralateral approach groups, in the study of mechanical effects on armpit-type LDH. Secondly, for periradicular-type LDH, the contralateral approach produced superior mechanical analysis findings than the ipsilateral approach. Finally, in terms of mechanical analysis findings, the contralateral approach revealed a considerable benefit over the ipsilateral approach for shoulder-type LDH. These experimental results are extremely important in guiding the selection of UBE surgical approaches.

**Fig. 9:**
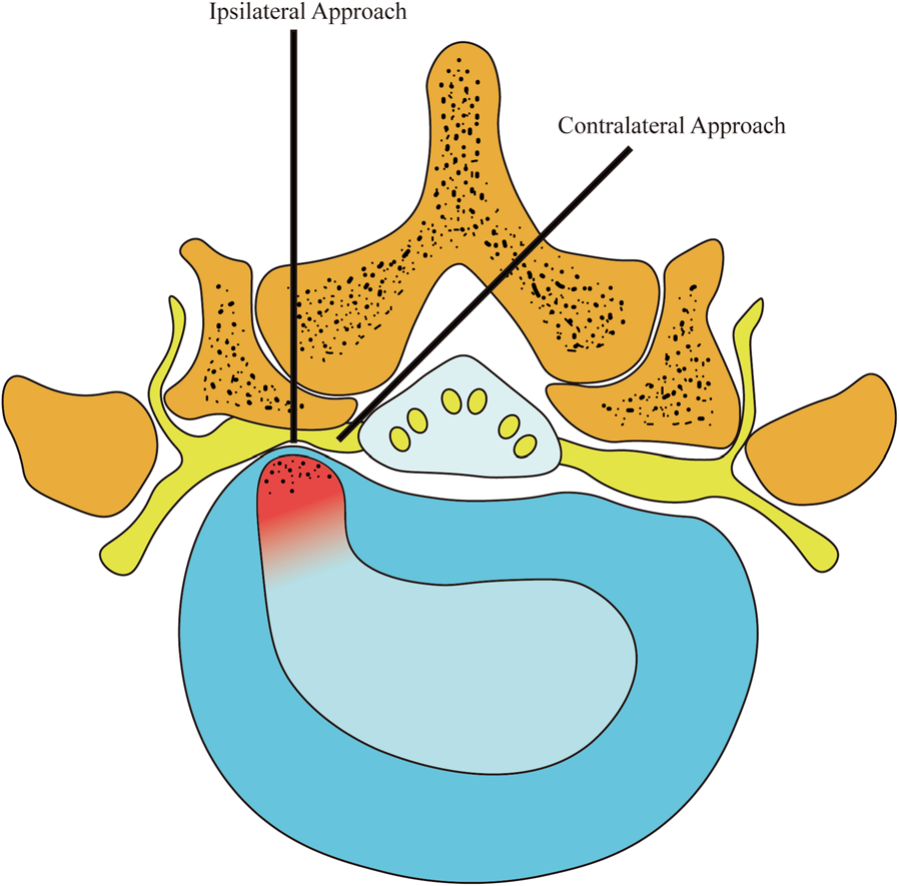
Demonstration diagrams of the two surgical approaches

The ipsilateral facet joint suffers unavoidable injury during ipsilateral approach decompression. The benefit of the contralateral approach, however, is the possibility to remove the diseased disc without severely injuring the facet joints [11, 29]. The main reason for this result is that, in ipsilateral approach surgery for periradicular-type LDH and shoulder-type LDH, even though the endoscope and surgical instruments are closest to the pathological site and the approach angle is close to 90°, the facet joints blocking the surgical pathway must be removed due to obstruction [27], as shown in Fig. 9, when performing an ipsilateral approach, facet joints become an obstruction that stands between the surgical approach and the ruptured disc. As a result, after UBE surgery using the ipsilateral approach, various degrees of injury occur to the facet joints and articular cartilage, resulting in a worsening of the overall mechanical qualities of the vertebrae [28]. However, when adopting the contralateral approach for periradicular-type LDH and shoulder-type LDH, the benefits of the contralateral approach may be used to directly access the target disc without damaging the facet joints. According to the literature, as compared to the contralateral approach, the ipsilateral approach has a higher postoperative resection rate of 22.6% of the articular eminence, and excessive resection of the eminence raises the fracture risk of the inferior articular eminence by 6% [29]. As a result, our mechanical analysis findings show that the contralateral approach in UBE surgery has a considerable advantage over the ipsilateral approach in preserving the integrity of the facet joints and articular cartilage, as well as maintaining the overall stability of the spine, it also decreases postoperative pain and discomfort, letting patients heal faster and lowering the chance of complications [30].

The likelihood of spinal degeneration increases after specific spinal interventions compared to the spine in its natural state. Some surgical procedures exacerbate spinal instability by removing bony structures and ligaments from the posterior column of the spine, indirectly accelerating the degeneration of neighbouring segments, which may lead to poorer spinal stability and reflect this problem through increased ROM [31, 32]. The ipsilateral approach to resection for shoulder-type LDH has a large area of facetectomy resection, which was also observed to have the greatest impact on ROM values and intervertebral discs in the trial results, and this can be seen as a major manifestation of the deterioration of lumbar spine stability [8].

In addition, for armpit-type LDH, there was little variation in biomechanical analyses between the ipsilateral and contralateral techniques. This is because our experimental design employed an idealized spinal model with absolutely symmetrical components. Furthermore, for armpit-type LDH, which protrudes at the junction of the midline and the fourth and fifth lumbar discs, the surgical paths in the ipsilateral and contralateral approaches are symmetric. As a result, there is minimal variation in the mechanical data between the two groups during flexion and extension. However, for right-side bending and rotation, the mechanical values in the ipsilateral approach were greater than those in the contralateral approach, and vice versa for left-side bending and rotation in the contralateral approach. The fundamental reason for this discrepancy is that in the ipsilateral approach, which is the entrance side, the bone damage occurs mostly on the right side of the vertebral lamina. As a result, the mechanical signs on the left side are worse during right-side bending and rotation of the spine. Similarly, with the contralateral approach, the predominant damage occurs on the left side of the lamina, resulting in poorer mechanical indicators on the right side during left-side bending and rotation.

**Fig. 10:**
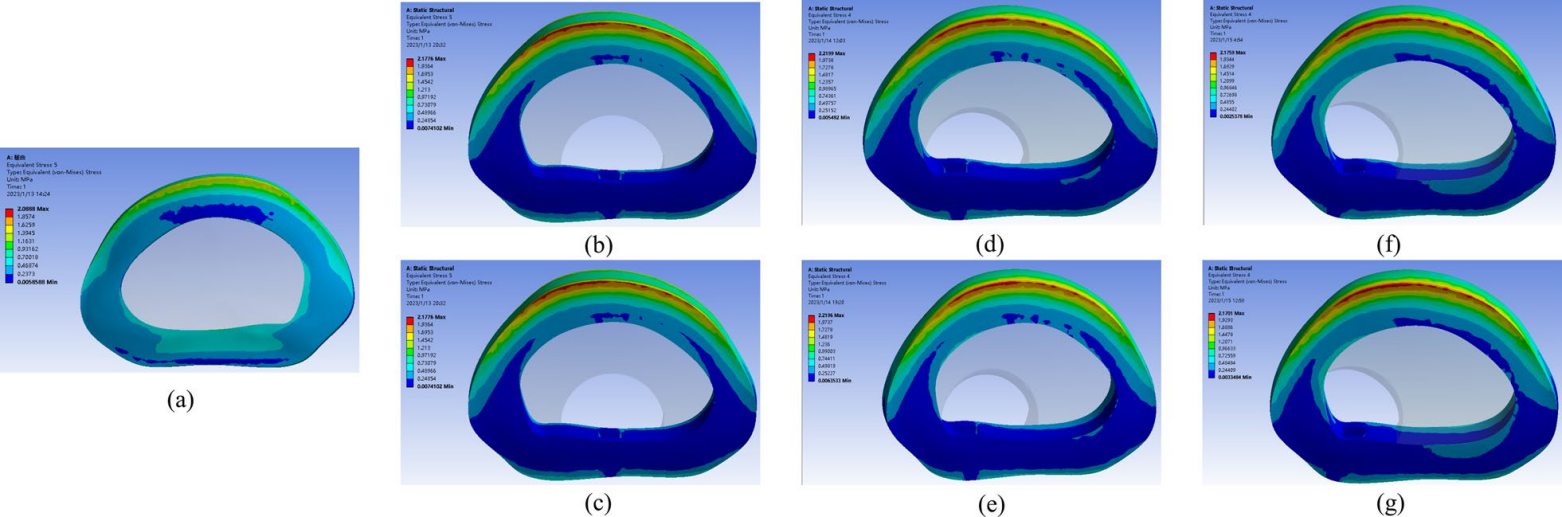
Nephogram of von Mises stress on the annulus in flexion condition on L4–L5. **a** Intact model. **b** Ipsilateral approach surgical excision of armpit-type LDH. **c** Contralateral approach surgical excision of armpit-type LDH. **d** Ipsilateral approach surgical excision of periradicular-type LDH. **e** Contralateral approach surgical excision of periradicular-type LDH. **f** Ipsilateral approach surgical excision of shoulder-type LDH. **g** Contralateral approach surgical excision of shoulder-type LDH

**Fig. 11:**
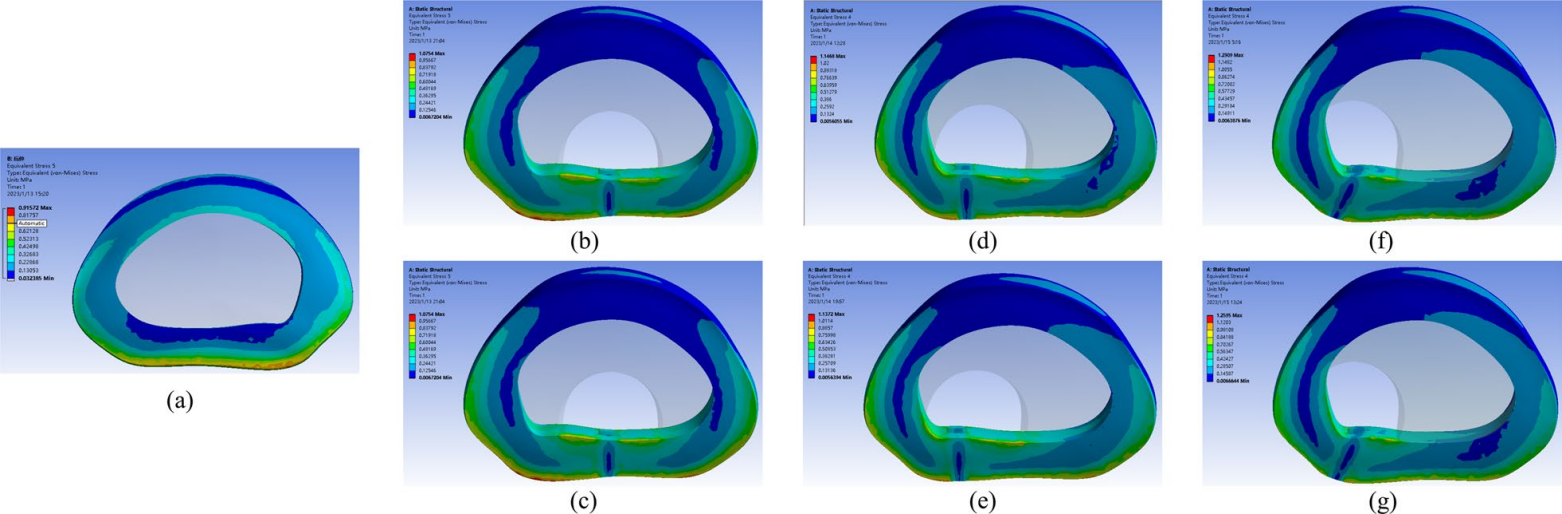
Nephogram of von Mises stress on the annulus in extension condition on L4–L5. **a** Intact model. **b** Ipsilateral approach surgical excision of armpit-type LDH. **c** Contralateral approach surgical excision of armpit-type LDH. **d** Ipsilateral approach surgical excision of periradicular-type LDH. **e** Contralateral approach surgical excision of periradicular-type LDH. **f** Ipsilateral approach surgical excision of shoulder-type LDH. **g** Contralateral approach surgical excision of shoulder-type LDH

Furthermore, when treating shoulder-type LDH with the ipsilateral approach, there is a higher disadvantage in terms of range of motion (ROM) values. The ipsilateral approach necessitates considerable excision of the afflicted side’s facet joints to completely expose the surgical route and the bulging disc, which has a major impact on spine stability [28]. It also worsens the damage to the articular cartilage and joint capsule, which are critical for keeping the annulus fibrosus loaded and the spine stable overall, damage to the articular cartilage and joint capsule can also lead to poor spinal stability and exacerbate fibrous ring damage [8, 33]. In addition, as shown in Figs. 10 and 11, the von Mises force distribution of the annulus fibrosus in flexion and posterior extension, damage to the articular eminence by the ipsilateral approach exacerbates the abnormal stress distribution in the annulus fibrosus, making the disc injury [20].

In summary, both the ipsilateral approach and the contralateral approach have advantages and disadvantages. The advantage of the ipsilateral approach is that the distance between the surgical approach and the ruptured disc is short, so it is easier for the operator to find the ruptured disc, but the disadvantage of this approach is that more facet joints need to be removed, which affects the stability of the patient’s lumbar spine after the operation, and it is not able to explore the lateral recesses area of the patient very well. The advantage of the contralateral approach is that there is less damage to the facet joints and soft tissues, and it is easier to detect the lesions in the lateral recesses of the patient, and there is more room for manoeuvring. However, the disadvantages of the contralateral approach are that the surgical path is too long, it is difficult to find the lesion site, and it is not suitable for novice surgeons [11, 27, 28].

Based on our findings, there is no significant difference in biomechanical effect between the ipsilateral and contralateral methods for armpit-type LDH. In terms of biomechanics, the contralateral approach is superior to the ipsilateral approach for periradicular-type LDH. To minimize excessive facet joint injury when doing decompression and to optimize patient results, we advocate employing the contralateral approach for shoulder-type LDH. The experimental results back up our findings.

Our experimental approach, however, has several drawbacks. We designed our spine model with a symmetrical lumbar spine model, with the limitation that it reduces the comprehensiveness of the study, and the impact of asymmetrical modelling should be explored in future studies. By lowering the contact sites of the ligament, the simulation of yellow ligament resection was accomplished, which may have impacted the experimental outcomes. Finally, our experimental design solely mimicked the mechanical analysis of the spine with ligament constraints and did not take into account the mechanical effect of the paraspinal muscles. As a consequence, the study’s findings had only a limited impact on real surgery outcomes.

## Conclusions

An ipsilateral or contralateral approach may be employed for armpit-type LDH to preserve as much as possible the integrity of the facet joints and to minimize lumbar instability following surgery, but we suggest a contralateral approach for periradicular-type and shoulder-type LDH.

## Data Availability

All the data of the manuscript are presented.

## Abbreviations

UBE: Unilateral bilateral endoscopy
LDH: Lumbar disc herniation
FEM: Finite element model
ROM: Range of motion
MRI: Magnetic resonance imaging
